# Calf Circumference as a Screening Tool for Cognitive Frailty in Community-Dwelling Older Adults: The Korean Frailty and Aging Cohort Study (KFACS)

**DOI:** 10.3390/jcm7100332

**Published:** 2018-10-08

**Authors:** Miji Kim, Min Jeong Jeong, Jinho Yoo, Da Young Song, Chang Won Won

**Affiliations:** 1 Department of Biomedical Science and Technology, College of Medicine, East-West Medical Research Institute, Kyung Hee University, Seoul 02447, Korea; mijiak@khu.ac.kr; 2 Elderly Frailty Research Center, Kyung Hee University, Seoul 02447, Korea; minjj914@khu.ac.kr (M.J.J.); hanasdy@naver.com (D.Y.S.); 3 Elderly Frailty Research Center, Department of Family Medicine, College of Medicine, Kyung Hee University, Seoul 02447, Korea; jhyoo153@naver.com

**Keywords:** calf circumference, cognitive frailty, frailty, aging, cohort study

## Abstract

The aim of this study was to examine calf circumference in relation to cognitive frailty in community-dwelling older adults. Cross-sectional analysis was performed on the first-year baseline data of 1559 adults aged 70–84 years enrolled in the Korean Frailty and Aging Cohort Study. The final analysis included 1221 adults who were non-dependent in terms of instrumental activities of daily living, who underwent frailty and cognitive function assessments. Physical frailty was defined using the Fried Frailty Index. Cognitive impairment was defined as a score 1.5 standard deviations below the age-, sex- and education-matched norms on any of four cognitive-function tests. The prevalence of cognitive frailty was 2.8% for men and 3.8% for women. After adjusting for potential confounders, in comparison to the “physically robust without cognitive impairment” group, the estimates of increased odds ratios (ORs) for low calf circumference (<32 cm) were much greater in the prefrail with cognitive impairment (OR 4.62, 95% confidence interval (CI): 2.02–10.61) and frail with cognitive impairment (OR 10.94, 95% CI: 2.87–41.68) groups in men but not in women. Low calf circumference was strongly related to cognitive frailty in men only, suggesting calf circumference can be used as an indicator of these outcomes.

## 1. Introduction

Frailty is common among people with the geriatric syndrome and results in adverse health outcomes including hospitalization, institutionalization, falls, functional disability, and mortality [1,2]. Adding cognitive impairment to the operational definition of the frail phenotype could improve its predictive validity with regard to adverse health outcomes [3]. Cognitive frailty is defined as the simultaneous presence of physical frailty and cognitive impairment [4]. In the Frailty Operative Definition Consensus Conference Project, experts agreed on the importance of a more comprehensive definition of frailty, to include both physical performance and cognition components [5]. Preclinical cognitive impairment was associated with earlier-onset frailty [6], with the two conditions being closely interrelated [7]. Physical frailty showed a relationship with an increased prevalence of cognitive impairment, and co-existing physical frailty and cognitive impairment conferred a greater risk of incident dementia [8]. A longitudinal, population-based study found that reversible cognitive frailty was a short- and long-term predictor of all-cause mortality and overall dementia in non-demented older individuals [9]. Hence, it is important that the tools potentially useful for identifying cognitively frail individuals capture the cognitive-frailty relationship, to inform future preventive and therapeutic strategies in older adults who may progress to dementia.

Loss of skeletal muscle mass and size, which occurs with aging, is a key component in the diagnosis of sarcopenia [10] and is associated with both physical frailty and cognitive impairment [11,12,13]. Calf circumference has been used as a simple proxy for skeletal muscle mass, sarcopenia, and nutritional status in clinical and community settings [14,15,16,17,18]. Calf circumference is related to frailty and functional performance [19] and is also a significant predictor of cognitive function [20]. Furthermore, calf circumference is a useful screening tool, being a simple, convenient and non-invasive measure. Calf circumference may be more closely associated with cognitive frailty than physical frailty and cognitive impairment independently, and thus it may be useful as a marker of cognitive frailty in older adults.

The aim of this study was to examine calf circumference in relation to cognitive frailty and to determine whether calf circumference can be used to screen for physical and cognitive frailty in community-dwelling older adults enrolled in the Korean Frailty and Aging Cohort Study (KFACS).

## 2. Materials and Methods

### 2.1. Study Population

**Figure 1:**
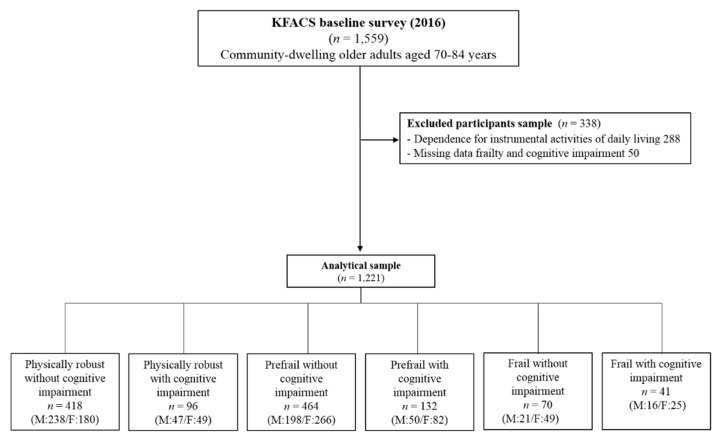
Flow chart of study participant selection.

The KFACS aims to identify diverse risk and preventive factors for the progression (and associated adverse outcomes) of frailty in community-dwelling older adults. The KFACS is a Korean multicenter longitudinal study, in which the baseline survey was conducted in 2016–2017. Sex- and age-stratified community residents aged 70 to 84 years, drawn from 10 medical centers in urban and rural regions nationwide, were eligible for participation in the study [21]. In total, there were 3014 participants in the baseline survey. Our analyses were performed using the baseline (i.e., 2016) data of 1559 KFACS participants. The final analysis included 1221 participants (570 men and 651 women), after excluding 288 participants who were dependent on others for instrumental activities of daily living (IADL) and 50 participants who had missing data for the outcomes of interest, such as frailty and cognitive impairment (Figure 1). The IADL were measured across 10 domains using the Korean IADL (K-IADL) instrument: decorating, housework, preparing meals, laundry, short outings, using transportation, shopping, handling money, using the telephone, and taking medicine.

### 2.2. Physical Frailty, Cognitive Impairment, and Cognitive Frailty

Physical frailty was defined using a modified version of the Fried Frailty Index, which covers five components of frailty [1]:

1. Unintentional weight loss: responding “yes” to the question: “In the last year, have you lost more than 4.5 kg unintentionally?” or exhibiting unintentional weight loss ≥5% of total body weight in the last year.
2. Weakness: maximal grip strength <26 kg for men and <18 kg for women, measured twice for each hand using a digital hand grip dynamometer (T.K.K. 5401; Takei Scientific Instruments Co, Ltd., Tokyo, Japan).
3. Self-reported exhaustion: responding “yes” to either of the following statements from the Center for Epidemiological Studies-Depression scale: “I felt that everything I did was an effort” and “I could not get going” on 3 or more days per week.
4. Slowness: 4-m gait speed <1.0 m/s, measured using an automatic timer (Gaitspeedometer Ver.1, Dynamicphysiology, Daejeon, Korea), with acceleration and deceleration phases of 1.5 m each. Gait speed was measured twice, and the mean values were used in the analysis.
5. Low physical activity: energy expenditure estimates (kcal/week) were calculated for various activities, and metabolic equivalent scores were derived using the International Physical Activity Questionnaire. Low physical activity level was defined as <494.65 kcal for men and <283.50 kcal for women, with these values corresponding to 20% of the total energy consumed in a population-based Korean survey of older adults from among the general population [22].

Total frailty scores (range: 0–5) were calculated by allocating a score of 1 to positive responses on each of the above five components. Participants with a score of 0 were classified as “robust”, a score of 1–2 as “prefrail”, and a score of 3–5 as “frail”.

Cognitive impairment was defined as a score 1.5 standard deviations (SDs) below the age-, sex- and education-matched Korean norms on any one of four cognitive-function tests: The Trail Making Test, Frontal Assessment Battery, Digit Span Backward, and the Word List Recall test [23]. Cognitive function was assessed using the Korean Version of the Consortium to Establish a Registry for Alzheimer’s Disease Assessment Packet [24,25], and the Korean version of the Frontal Assessment Battery [26]. The International Academy on Nutrition and Aging and the International Association of Gerontology and Geriatrics group proposed the definition of cognitive impairment using the Clinical Dementia Rating (global CDR score of 0.5), excluding concurrent dementia [4]. Because the CDR is not often available in epidemiologic studies and is difficult to implement, an alternative is required. Furthermore, mild cognitive impairment (MCI) may not be an appropriate component in the diagnostic criteria for cognitive frailty due to the lack of benefits for dementia prevention [27]. In general, scores on neuropsychological tests for individuals with MCI are 1.0 to 1.5 SDs below the mean for age- and education-matched control subjects [28]. A consensus from the Subjective Cognitive Decline Initiative Working Group proposed research criteria for cognitive-function testing for pre-MCR subjective cognitive decline, which was defined as less than or equal to 1.5 standard deviations from age-, sex-, and education-adjusted norms on standardized cognitive tests [29,30]. In recent years, epidemiologic studies have used this definition of cognitive impairment [31,32].

Cognitive frailty was defined as the presence of both physical frailty and cognitive impairment. Based on the degree of physical frailty and cognitive impairment, participants were classified into one of six groups: physically robust without cognitive impairment, physically robust with cognitive impairment, prefrail without cognitive impairment, prefrail with cognitive impairment, frail without cognitive impairment, or frail with cognitive impairment. The percentages of the participants in each group were 34.2%, 7.9%, 38.0%, 10.8%, 5.7%, and 3.4%, respectively (Figure 1).

### 2.3. Calf Circumference

Calf circumference was measured with the participant standing upright and the legs slightly apart. A measuring tape was positioned around the calf at the point of maximum circumference; subcutaneous tissue was not compressed [15]. Our previous study suggested a cut-off value of 32 cm for diagnosing low muscle mass and sarcopenia based on the Asian Working Group for Sarcopenia definition for community-dwelling older adults [15].

### 2.4. Other Measurements

An in-person interview and health examination were performed. Participants provided information on smoking status, alcohol consumption, education level, marital status, and medical history and treatment, via a questionnaire. Comorbid status was determined by the presence of at least one of the following diseases: myocardial infarction, congestive heart failure, peripheral vascular disease, cerebrovascular disease, chronic obstructive pulmonary disease, rheumatoid arthritis, ulcers, mild liver disease, diabetes mellitus, diabetes with complications, hemiplegia, moderate or severe renal disease, any tumor, moderate or severe liver disease, leukemia, lymphoma, or acquired immune deficiency syndrome. Body mass index (BMI) was calculated as weight (kg) divided by height squared (m^2^). Body composition was measured using dual energy X-ray absorptiometry (Lunar; GE Healthcare, Madison, WI, USA; and Hologic DXA; Hologic Inc., Bedford MA, USA) and bioelectrical impedance analysis (InBody 720; InBody Co., Ltd., Seoul, Korea and X-SCAN PLUS II; Jawon Medical Inc., Seoul, Korea). Appendicular skeletal muscle mass (ASM; kg), that is, the sum of the lean soft-tissue mass in both extremities, was measured. The ASM index was calculated as the ASM divided by height squared (m^2^). The definition of low muscle mass used herein was that of the Asian Working Group for Sarcopenia: <7.0 kg/m^2^ for men and <5.4 kg/m^2^ for women on dual energy X-ray absorptiometry and <7.0 for men and <5.7 kg/m^2^ for women on bioelectrical impedance analysis [33]. The Short Physical Performance Battery (SPPB) consists of three standing balance measures (tandem, semi-tandem, and side-by-side stands), five chair-stand time measures, and assessment of typical gait speed. Each test is scored from 0 to 4, based on the normative scores of the Established Population for Epidemiologic Studies of the Elderly [34]. The scores are summed to obtain a total score, ranging from 0 to 12 [34,35]. In the Timed Up And Go (TUG) test, participants were asked to rise from an armchair of standard height; a 3-m distance was marked on the floor in front of the chair [36]. The starting position was sitting with the hands resting on the arms of the chair. The participants crossed the line before turning around and walking back to, and sitting down on, the chair. They were instructed to perform the TUG test at their own comfortable and safe walking pace. The TUG test started as soon as the participant’s back ceased to be in contact with the back of the chair, and stopped when their buttocks recontacted the seat of the chair.

### 2.5. Ethics

The KFACS protocol was approved by the Institutional Review Board (IRB) of the Clinical Research Ethics Committee of Kyung Hee University Medical Center, Seoul, Korea, and all participants provided written informed consent (IRB number: 2015-12-103). This study was exempt from review by the IRB (IRB number: 2018-05-095).

### 2.6. Statistical Analysis

The data are presented as means ± SD or as numbers (percentages). The relationships among participant characteristics and frailty and cognitive function indices were analyzed by analysis of variance (ANOVA) and chi-square tests. We calculated geometric means (standard error), estimates, and 95% confidence intervals (CIs) using a generalized linear model to assess calf circumference according to physical frailty and cognitive impairment status. In the secondary analysis, we used multivariable logistic regression models with Firth’s penalized likelihood method to address issues of small sample size [37]. Odds ratios (95% CIs) for low calf circumference (<32 cm) were estimated by cognitive frailty status. Models were adjusted for age (continuous), total alcohol consumption (never or ever drinker), years of education (0–6 or ≥7), number of comorbidities (0 or ≥1), number of medications (0, 1–4, or ≥5), and body mass index (≤23, 23–24.9, 25–26.9, ≥27). All analyses were conducted using SPSS software (ver. 23.0; SPSS Inc., Chicago, IL, USA) and SAS version 9.4 (SAS Institute, Inc., Cary, NC, USA). Significance was set at a two-sided *p*-value of <0.05.

## 3. Results

Table 1 presents the baseline characteristics of the male participants according to physical frailty and cognitive impairment status. The mean age of the men was 76.4 years; 2.8% were both physically frail and cognitively impaired, that is, cognitively frail. Compared with the physically robust without cognitive impairment group, those who were prefrail or frail, with or without cognitive impairment, were older, less educated, less likely to be married and had a smaller calf circumference. Table 2 presents the baseline characteristics of the female participants. The mean age of the women was 75.7 years; 3.8% were cognitively frail, and 12.6% were cognitively prefrail.

Table 3 and Table 4 show the generalized linear model estimates and 95% CIs for calf circumference according to physical frailty and cognitive impairment status in men and women. After adjusting for potential confounders, including age, sex, alcohol consumption, years of education, marital status, comorbidities, medications, and body mass index (Model 3), significant decreases in calf circumference were found for the physically robust with cognitive impairment group (estimate (β) = −0.682, 95% CI = −1.352; −0.031), the prefrail without cognitive impairment group (β = −0.745, 95% CI = −1.150; −0.340), the prefrail with cognitive impairment group (β = −0.932, 95% CI = −1.589; −0.275), the frail without cognitive impairment group (β = −1.229, 95% CI = −2.274; −0.324), and the frail with cognitive impairment group (β = −2.471, 95% CI = −3.566; −1.377), as compared with the physically robust without cognitive impairment group (all <0.05). In women (Model 3), significant decreases were found in calf circumference for the prefrail without cognitive impairment group (β = −0.408, 95% CI = −1.806; −0.011), the prefrail with cognitive impairment group (β = −0.681, 95% CI = −1.241; −0.121), and the frail without cognitive impairment group (β = −1.104, 95% CI = −1.799; −0.409) as compared with the physically robust without cognitive impairment group (all <0.05). However, the physically robust with cognitive impairment and frail with cognitive impairment groups did not show significant associations with decreased calf circumference.

**Figure 2:**
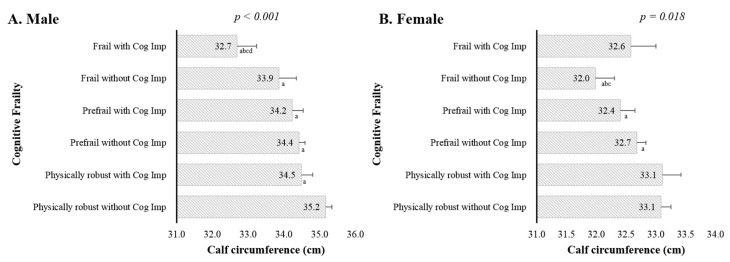
(**A**) male; (**B**) female. Geometric mean (±SE) calf circumference according to physical frailty and cognitive impairment status. The multivariate analyses were adjusted for Model 3 (age, alcohol, years of education, comorbidity, medication, and body mass index). *p*-value from test for linear trend. SE = standard error; Cog Imp = cognitive impairment. Differences determined by post-hoc analysis were considered significant at *p* < 0.05. ^a^ Significantly different from physically robust without Cog Imp group. ^b^ Significantly different from physically robust with Cog Imp group. ^c^ Significantly different from prefrail without Cog Imp group. ^d^ Significantly different from prefrail with Cog Imp group.

**Figure 3:**
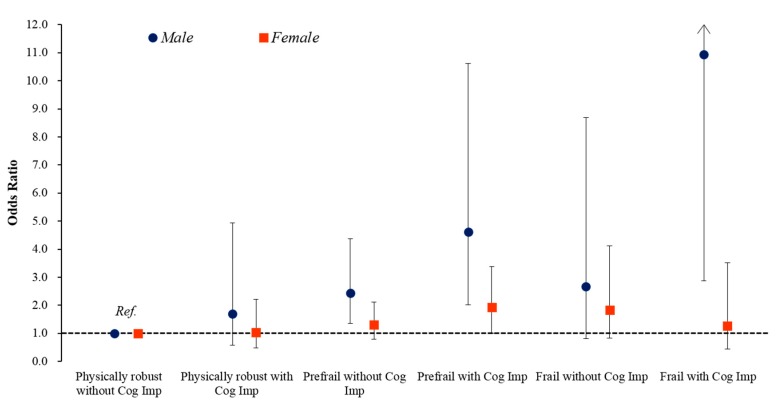
Association between low calf circumference (<32 cm) and cognitive frailty status. Odds ratios (95% confidence intervals) were calculated by Firth’s penalized likelihood of logistic regression after adjustment for age, alcohol, years of education, comorbidity, medication, and body mass index.

Figure 2 shows that the geometric mean calf circumference after adjusting for potential confounding variables (Model 3) showed a significant decreasing trend across the six groups, from the physically robust without cognitive impairment group to the frail with cognitive impairment group in men (35.2, 34.5, 34.4, 34.2, 33.9, 32.7, respectively, *p* for trend < 0.001). In women, this pattern was not seen (33.1, 33.1, 32.7, 32.4, 32.0, 32.6, respectively, *p* for trend = 0.018). In the post-hoc analysis, the geometric mean calf circumference of the men was significantly lower in the frail with cognitive impairment group compared with the physically robust without cognitive impairment, physically robust with cognitive impairment, prefrail without cognitive impairment, and prefrail with cognitive impairment groups (all *p* < 0.5); this pattern was not seen in women. Figure 3 shows the association between low calf circumference (<32 cm) and cognitive frailty status. The percentages with a low calf circumference were 19.5% for mean and 37.6% for women. The odds ratios (ORs) for low calf circumference are presented by cognitive frailty status. In comparison to the “physically robust without cognitive impairment” group, the estimates of increased ORs for low calf circumference were much greater in the prefrail with cognitive impairment and frail with cognitive impairment groups in men. After adjusting for potential confounding variables (Model 3), the ORs (95% CI) were 1.69 (0.58–4.94) for physically robust with cognitive impairment, 2.44 (1.36–4.37) for prefrail without cognitive impairment, 4.62 (2.02–10.61) for prefrail with cognitive impairment, 2.66 (0.81–8.68) for frail without cognitive impairment, and 10.94 (2.87–41.68) for frail with cognitive impairment. However, there were no associations between low calf circumference and cognitive frailty status in women. When compared with the physically robust without cognitive impairment reference group, after controlling for potential confounders (Model 3), the ORs (95% CI) were 1.04 (0.48–2.22) for physically robust with cognitive impairment, 1.30 (0.80–2.11) for prefrail without cognitive impairment, 1.93 (1.00–3.71) for prefrail with cognitive impairment, 1.83 (0.82–4.11) for frail without cognitive impairment, and 1.26 (0.45–3.51) for frail with cognitive impairment.

## 4. Discussion

In our community-based sample of older adults, we found a significant decreasing trend in calf circumference across the six study groups, ranging from the physically robust without cognitive impairment to the physically frail with cognitive impairment group, in men only. Furthermore, we demonstrated that low calf circumference can be used to screen for prefrailty, cognitive prefrailty, and cognitive frailty in men, but not in women. Our findings underscore the utility of combining physical frailty and cognitive impairment indices and imply that calf circumference can serve as a simple screening tool for potential early detection of elderly men at risk of cognitive frailty. In the present analyses, no association was found between low calf circumference and cognitive frailty among elderly women. To the best of our knowledge, this is the first study to investigate the relationship between calf circumference and cognitive frailty in community-dwelling older adults.

The prevalence of co-existing physical frailty and cognitive impairment was approximately 3.4% among our sample of older Koreans, and was significantly higher in women than in men. Similar to these results, Shimada et al. reported a prevalence of physical frailty (according to the Fried Frailty Index) accompanied by mild cognitive impairment of 2.7% in a sample of 5104 Japanese community-dwelling elderly adults (aged ≥ 65 years) [38]. However, in a systematic review, the prevalence of cognitive frailty, ranging from 1.0% to 39.7%, depended on the population characteristics and definition used [39]. Cognitive frailty has been associated with high risks of disability and dementia, poor quality of life, and death in the community setting [3,8,40,41,42].

Calf circumference has been used as a simple proxy marker for skeletal muscle mass, sarcopenia, and nutritional status in clinical and community settings [14,15,16,17,18]. Furthermore, it has been shown to be effective in predicting subsequent disability, an emerging need for care, and mortality in older adults [43,44,45]. On analyzing a representative sample of older US adults (age range: 60–84 years) according to four anthropometric parameters (waist, arm, thigh, and calf circumferences), only calf circumference showed a significant (inverse) association with disability [44]. In the Taiwan Longitudinal Survey on Aging Study [45], calf circumference was a better predictor of an emerging need for care in older adults compared to BMI.

In our study, we found a significant association between calf circumference and physical frailty in both sexes, consistent with the findings of the Aging and Longevity Study [19]. In that study, after adjusting for potential confounders, the Fried Frailty Index was significantly lower in participants with a larger versus smaller calf circumference (1.66 vs. 2.17, respectively) among a population of older adults (aged ≥ 80 years). In another study, there was a close relationship between calf circumference and ASM [46]. Mechanistically, muscle mass atrophy is a major factor in the pathogenesis of frailty [47], whereas calf circumference is a marker of the reduced muscle mass that characterizes sarcopenia and is related to physical function [14,15]. Nutritional status may be poor in frail older adults [48], and calf circumference can serve as a proxy for monitoring nutritional status in older populations [49]. In summary, calf circumference may be useful for monitoring physical frailty in older community-dwelling adults in clinical and research settings.

Age-related decline in cognitive function is a key aspect of dementia, and is also important for early detection of cognitive impairment in older community-dwelling adults. Body composition may serve as a modifiable risk factor of cognitive impairment. In a meta-analysis, sarcopenia and low muscle mass were independently associated with cognitive impairment [12]. Nishiguchi et al. found that both cognitive impairment and sarcopenia were associated with frailty [50]. In a population-based cross-sectional study, increased muscle mass and a lower level of adipose tissue were associated with superior cognitive function in older adults aged ≥60 years [20]. Specifically, these studies showed that a larger calf circumference was associated with superior global cognition, memory, attention, information processing, and verbal learning. The present study showed that low calf circumference was most strongly related to cognitive impairment in community-dwelling Korean elderly men. Unlike in men, the lowest calf circumference was not seen in the cognitively frail group of women. Furthermore, in our subsequent analysis, there was no association between low calf circumference (<31 cm) and cognitive frailty status in women (data not shown). In a previous study, a calf circumference of 31 cm was considered the cut-off point for low muscle mass in community-dwelling older women [46]. One possible explanation for this difference is that the strength of the relationship between cognitive function and (age-related) decreased muscle mass differs between men and women: cognitive frailty was associated with low muscle mass (87.5%) in men, but not in women (32.0%). This is in line with previous studies regarding sex-specific differences in the relationship between low muscle mass and impaired cognitive functioning [51,52]. A large epidemiological study reported no association between low muscle mass and cognitive impairment after adjusting for potential confounders in a sample of 3025 women aged ≥75 years [51]. A Chinese study showed a significant relationship between cognitive impairment and low muscle mass (whole-body muscle mass and ASM) among community-dwelling men aged ≥65 years, but not in women [52]. Similarly, inverse associations between fat and dementia in women may be related to the regulation of female hormones [11]. In that study, higher percentages of subcutaneous abdominal and thigh fat were associated with a lower likelihood of dementia in women only. Longitudinal studies are warranted to elucidate further the mechanism underlying the association between calf circumference and cognitive impairment.

Our study had some limitations. First, its cross-sectional design did not allow evaluation of any cause–effect relationship between calf circumference and cognitive frailty. Second, our participants were community-dwelling older adults, recruited in a research setting; therefore, our results may not be generalized to other settings and populations. To confirm further the association between calf circumference and cognitive impairment, prospective studies are needed. We plan to conduct a follow-up longitudinal study on the predictive validity of calf circumference for development of cognitive frailty. Despite some limitations, this study benefitted from including a nationally representative sample of community-dwelling elderly Korean adults.

## 5. Conclusions

This study showed that low calf circumference was strongly related to cognitive frailty in community-dwelling Korean elderly men, for whom calf circumference can therefore be used as an indicator of cognitive frailty. Longitudinal studies are needed to determine the association between calf circumference and cognitive frailty over longer periods and sex-based differences.

## Figures and Tables

**Table 1:** Characteristics of the male participants (*n* = 570) according to physical frailty and cognitive impairment status.

Variable	Physically Robust without Cog Imp (*n* = 238)		Physically Robust with Cog Imp (*n* = 47)		Prefrail without Cog Imp (*n* = 198)		Prefrail with Cog Imp (*n* = 50)		Frail without Cog Imp (*n* = 21)		Frail with Cog Imp (*n* = 16)		*p*-Value
Age (years)	75.7	(3.7)	76.1	(3.7)	76.7	(4.1)	77.4	(3.6)	78.8	(3.8)	78.3	(3.6)	<0.001
BMI (kg/m^2^)	24.0	(2.7)	25.0	(2.4)	23.9	(3.1)	23.9	(3.1)	23.2	(3.6)	21.2	(2.5)	0.001
Smoking status													
Never	50	(21.0)	15	(31.9)	37	(18.7)	10	(20.0)	6	(28.6)	2	(12.5)	0.363
Ever	188	(79.0)	32	(68.1)	161	(81.3)	40	(80.0)	15	(71.4)	14	(87.5)	
Alcohol (servings per week) ^a^													
Never	62	(26.1)	15	(32.6)	68	(34.3)	25	(50.0)	9	(42.9)	7	(43.8)	0.018
Ever	176	(74.0)	31	(67.4)	130	(65.7)	25	(50.0)	12	(57.1)	9	(56.2)	
Education (years)													
0–6	56	(23.5)	11	(23.4)	62	(31.3)	16	(32.0)	11	(52.4)	8	(50.0)	0.016
≥7	182	(76.5)	36	(76.6)	136	(68.7)	34	(68.0)	10	(47.6)	8	(50.0)	
Marital status													
Married	218	(91.6)	40	(85.1)	172	(86.9)	42	(84.0)	18	(85.7)	11	(68.8)	0.077
Unmarried, divorced, widowed, or separated	20	(8.4)	7	(14.9)	26	(13.1)	8	(16.0)	3	(14.3)	5	(31.2)	
SPPB score ^a^	11.4	(0.9)	11.1	(1.3)	11.0	(1.2)	10.6	(1.5)	10.0	(2.3)	9.1	(2.2)	<0.001
Timed Up and Go test													
≤10 s	165	(69.3)	25	(53.2)	96	(48.5)	17	(34.0)	2	(9.5)	1	(6.2)	<0.001
>10 s	73	(30.7)	22	(46.8)	102	(51.5)	33	(66.0)	19	(90.5)	15	(93.8)	
ASM/height^2^ (kg/m^2^) ^a^	7.2	(0.9)	7.4	(0.8)	7.1	(1.0)	7.2	(0.9)	7.1	(1.0)	6.2	(0.7)	<0.001
Normal muscle mass	139	(58.9)	34	(72.3)	98	(49.8)	27	(54.0)	13	(61.9)	2	(12.5)	0.001
Low muscle mass	97	(41.1)	13	(27.7)	99	(50.2)	23	(46.0)	8	(38.1)	14	(87.5)	
Number of comorbidities													
0	160	(67.2)	36	(76.6)	103	(52.0)	28	(56.0)	6	(28.6)	9	(56.2)	<0.001
≥1	78	(32.8)	11	(23.4)	95	(48.0)	22	(44.0)	15	(71.4)	7	(43.8)	
Number of medications ^a^													
0	54	(22.7)	12	(25.5)	31	(15.7)	4	(8.0)	0	(0.0)	1	(6.2)	<0.001
1–4	112	(47.1)	26	(55.3)	88	(44.4)	20	(40.0)	7	(33.3)	7	(43.8)	
≥5	72	(30.2)	9	(19.2)	79	(39.9)	26	(52.0)	14	(66.7)	8	(50.0)	
Calf circumference (cm)	34.8	(2.4)	34.7	(2.8)	33.8	(2.8)	33.6	(2.8)	32.6	(3.4)	30.5	(2.4)	<0.001

Note: Values are means (±SD) or numbers (percentages). *p*-values were calculated by ANOVA for continuous variables and by chi-square or Fisher’s exact test for categorical variables. ASM, appendicular skeletal mass; BMI, body mass index; Cog Imp, cognitive impairment; SPPB, Short Physical Performance Battery. ^a^ missing values.

**Table 2:** Characteristics of the female participants (*n* = 651) according to physical frailty and cognitive impairment status.

Variable	Physically Robust without Cog Imp (*n* = 180)		Physically Robust with Cog Imp (*n* = 49)		Prefrail without Cog Imp (*n* = 266)		Prefrail with Cog Imp (*n* = 82)		Frail without Cog Imp (*n* = 49)		Frail with Cog Imp (*n* = 25)		*p*-Value
Age (years)	74.5	(3.4)	75.2	(3.6)	75.7	(4.0)	76.0	(3.3)	78.1	(4.3)	78.5	(3.2)	<0.001
BMI (kg/m^2^)	24.9	(3.0)	24.0	(2.2)	24.5	(2.9)	25.0	(2.7)	24.3	(3.3)	24.9	(3.9)	0.704
Smoking status													
Never	177	(98.3)	46	(93.9)	259	(97.4)	81	(98.8)	47	(95.9)	24	(96.0)	0.360
Ever	3	(1.7)	3	(6.1)	7	(2.6)	1	(1.2)	2	(4.1)	1	(4.0)	
Alcohol (servings per week) ^a^													
Never	103	(58.2)	34	(69.4)	165	(62.3)	47	(58.8)	38	(77.6)	15	(60.0)	0.176
Ever	74	(41.8)	15	(30.6)	100	(37.7)	33	(41.3)	11	(22.4)	10	(40.0)	
Education (years)													
0–6	84	(46.7)	29	(59.2)	150	(56.4)	62	(75.6)	43	(87.8)	21	(84.0)	<0.001
≥7	96	(53.3)	20	(40.8)	116	(43.6)	20	(24.4)	6	(12.2)	4	(16.0)	
Marital status													
Married	85	(47.2)	20	(40.8)	123	(46.2)	30	(36.6)	21	(42.9)	8	(32.0)	0.439
Unmarried, divorced, widowed, or separated	95	(52.8)	29	(59.2)	143	(53.8)	52	(63.4)	28	(57.1)	17	(68.0)	
SPPB score ^a^	11.3	(1.0)	11.2	(1.1)	10.8	(1.2)	10.3	(1.5)	9.4	(1.8)	9.0	(1.6)	<0.001
Timed Up and Go test													
≤10 s	125	(69.4)	34	(69.4)	134	(50.4)	25	(30.5)	3	(6.1)	0	(0.0)	<0.001
>10 s	55	(30.6)	15	(30.6)	132	(49.6)	57	(69.5)	46	(93.9)	25	(100.0)	
ASM/height^2^ (kg/m^2^) ^a^	6.1	(0.9)	6.2	(0.9)	6.0	(0.9)	5.9	(0.8)	6.0	(0.9)	6.0	(1.1)	0.704
Normal muscle mass	141	(78.3)	43	(87.8)	206	(77.4)	54	(65.9)	33	(67.4)	17	(68.0)	0.034
Low muscle mass	39	(21.7)	6	(12.2)	60	(22.6)	28	(34.1)	16	(32.7)	8	(32.0)	
Number of comorbidities													
0	124	(68.9)	39	(79.6)	179	(67.3)	48	(58.5)	27	(55.1)	18	(72.0)	0.079
≥1	56	(31.1)	10	(20.4)	87	(32.7)	34	(41.5)	22	(44.9)	7	(28.0)	
Number of medications ^a^													
0	33	(18.3)	11	(22.5)	29	(10.9)	14	(17.1)	1	(2.0)	6	(24.0)	<0.001
1–4	107	(59.4)	28	(57.1)	162	(60.9)	35	(42.7)	19	(38.8)	12	(48.0)	
≥5	40	(22.2)	10	(20.4)	75	(28.2)	33	(40.2)	29	(59.2)	7	(28.0)	
Calf circumference (cm)	33.1	(2.7)	32.7	(2.1)	32.6	(2.6)	32.3	(2.9)	31.1	(3.2)	31.7	(3.0)	<0.001

Note: Values are means (± SD) or numbers (percentages). *p*-values were calculated by ANOVA for continuous variables and by chi-square or Fisher’s exact test for categorical variables. ASM, appendicular skeletal mass; BMI, body mass index; Cog Imp, cognitive impairment; SPPB, Short Physical Performance Battery. ^a^ missing values.

**Table 3:** Generalized linear model estimates for calf circumference (cm) according to physical frailty and cognitive impairment status in men.

Variable		Unadjusted			Model 1			Model 2			Model 3	
	Estimate	95% CI	*p*-Value	Estimate	95% CI	*p*-Value	Estimate	95% CI	*p*-Value	Estimate	95% CI	*p*-Value
Cognitive frailty status												
Physically robust without Cog Imp	*Ref.*			*Ref.*			*Ref.*			*Ref.*		
Physically robust with Cog Imp	−0.139	−0.982, 0.703	0.746	−0.109	−0.947, 0.729	0.789	−1.116	−0.956, 0.723	0.786	−0.682	−1.352, −0.013	0.046
Prefrail without Cog Imp	−0.946	−1.472, −0.457	0.000	−0.885	−1.393, −0.377	0.001	−0.803	−1.310, −0.296	0.002	−0.745	−1.150, −0.340	<0.001
Prefrail with Cog Imp	−1.243	−2.064, −0.422	0.003	−1.107	−1.930, −0.285	0.008	−0.959	−1.783, −1.134	0.023	−0.932	−1.589, −0.275	0.005
Frail without Cog Imp	−2.182	−3.383, −0.980	0.000	−1.945	−3.152, −0.738	0.002	−1.697	−2.905, −0.488	0.006	−1.299	−2.274, −0.324	0.009
Frail with Cog Imp	−4.301	−5.664, −2.938	0.000	−4.098	−5.462, −2.735	0.000	−3.862	−5.224, −2.501	0.000	−2.471	−3.566, −1.377	<0.001

Note: CI, 95% confidence intervals; Cog Imp, cognitive impairment; *Ref.*, reference. Model 1: adjusted for age (years; continuous). Model 2: adjusted for age (years; continuous), alcohol consumption (never, ever), and years of education (0–6, ≥7). Model 3: adjusted for age (years; continuous), alcohol consumption (never, ever), years of education (0–6, ≥7), comorbidities (0, ≥1), medications (0, 1–4, ≥5), and body mass index (≤23, 23–24.9, 25–26.9, ≥27).

**Table 4:** Generalized linear model estimates for calf circumference (cm) according to physical frailty and cognitive impairment status in women.

Variable		Unadjusted			Model 1			Model 2			Model 3	
	Estimate	95% CI	*p*-Value	Estimate	95% CI	*p*-Value	Estimate	95% CI	*p*-Value	Estimate	95% CI	*p*-Value
Cognitive frailty status										*Ref.*		
Physically robust without Cog Imp	*Ref.*			*Ref.*			*Ref.*					
Physically robust with Cog Imp	−0.370	−1.216, 0.476	0.391	−0.282	−1.116, 0.551	0.507	−0.232	−1.071, 0.606	0.587	0.021	−1.636, 0.677	0.951
Prefrail without Cog Imp	−0.493	−1.000, 0.013	0.056	−0.331	−0.835, 0.172	0.197	−0.301	−0.809, 0.280	0.246	−0.408	−1.806, −0.011	0.044
Prefrail with Cog Imp	−0.842	−1.542, −0.143	0.018	−0.642	−1.336, 0.052	0.070	−0.515	−1.229, 0.198	0.157	−0.681	−1.241, −0.121	0.017
Frail without Cog Imp	−2.001	−2.847, −1.115	0.000	−1.535	−2.391, −0.679	0.000	−1.362	−2.240, −0.484	0.002	−1.104	−1.799, −0.409	0.002
Frail with Cog Imp	−1.378	−2.499, −0.258	0.016	−0.859	−1.984, 0.266	0.135	−0.712	−1.849, 0.425	0.220	−0.509	−1.397, 0.379	0.261

Note: CI, 95% confidence intervals; Cog Imp, cognitive impairment; *Ref.*, reference. Model 1: adjusted for age (years; continuous). Model 2: adjusted for age (years; continuous), alcohol consumption (never, ever), and years of education (0–6, ≥7). Model 3: adjusted for age (years; continuous), alcohol consumption (never, ever), years of education (0–6, ≥7), comorbidities (0, ≥1), medications (0, 1–4, ≥5), and body mass index (≤23, 23–24.9, 25–26.9, ≥27).
